# Supplementary material for: Determining Minimum Energy Conical Intersections by Enveloping the Seam: Exploring Ground and Excited State Intersections in Coupled Cluster Theory
Source: J Phys Chem Lett. 2025 Jan 7;16(2):561–7. doi: 10.1021/acs.jpclett.4c03274 (PMC11748164; doi:10.1021/acs.jpclett.4c03274)
Supplement: Supplementary file 1 — jz4c03274_si_001.pdf [file jz4c03274_si_001.pdf]

Supporting Information for "Determining minimum energy conical intersections by enveloping the seam: exploring ground and excited state intersections in coupled cluster theory"

Sara Angelico, Eirik F. Kjønstad, and Henrik Koch*

Department of Chemistry, Norwegian University of Science and Technology, NTNU, 7491 Trondheim, Norway

E-mail: henrik.koch@ntnu.no

Convergence of the algorithm: S_1/S_2 MECIs for uracil

To analyze the convergence of the algorithm, we study the S_1/S_2 ε-MECI in uracil using EOM-SCCSD. In Fig. 1 and 2, we compare the convergence properties of a stepwise optimization using the tube algorithm with the gradient projection method. All optimizations were converged to $3 \cdot 10^{-4}$ au in the maximum element of the gradient and $1.2 \cdot 10^{-4}$ au in the root mean square (rms) of the gradient. For the tube algorithm, we performed a first optimization with $\varepsilon = 0.27$ eV and used the converged geometry as an initial guess for a second optimization with $\varepsilon = 0.027$ eV.

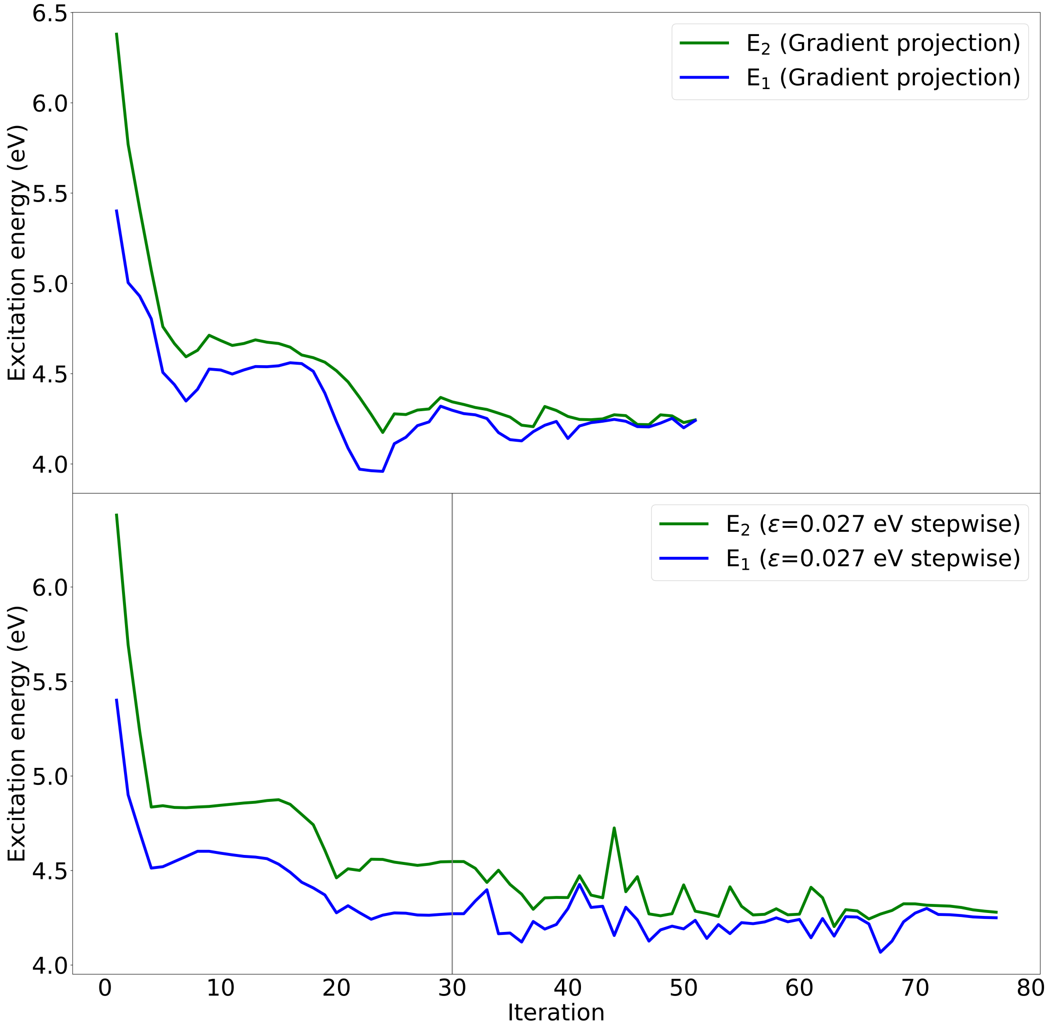

Figure 1: Excitation energies of S_1 and S_2 over the number of iterations for an optimization using the gradient projection method (top) and a stepwise optimization using the tube algorithm (bottom). The grey line indicates the first convergence of the stepwise tube algorithm with $\varepsilon = 0.27$ eV.

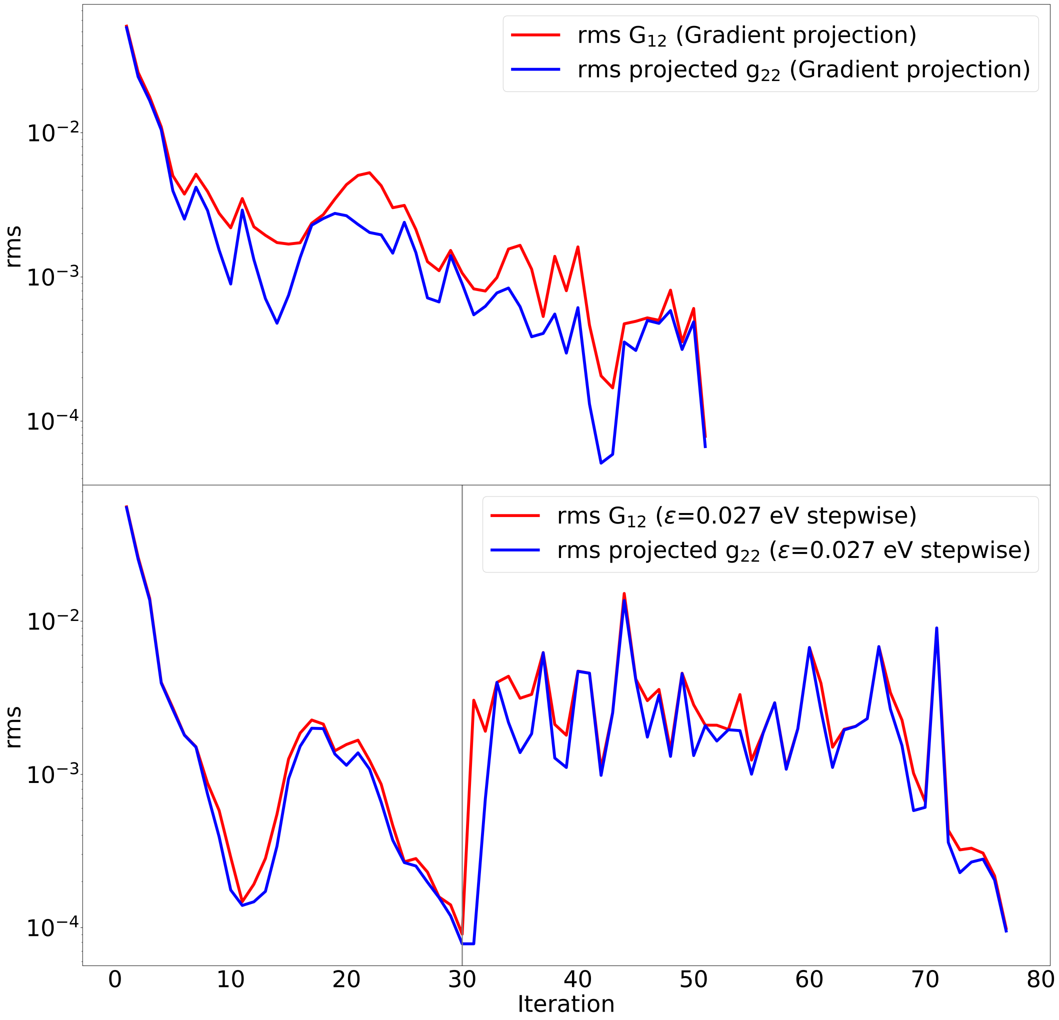

Figure 2: Root mean square displacement (rms) of the total gradient G_{12} and of the projection of g_{22} along the tube over the number of iterations for an optimization using the gradient projection method (top) and a stepwise optimization using the tube algorithm (bottom). The grey line indicates the first convergence of the stepwise tube algorithm with $\varepsilon = 0.27$ eV.

Comparison of EOM-CCSD/EOM-SCCSD geometries for the S_1/S_2 MECIs for uracil (ε=0.027 eV)

Table 1: Comparison of internal coordinates for the S_1/S_2 ε-MECI for uracil determined with $\varepsilon = 0.027$ eV and using CCSD or SCCSD. Distances are given in angstrom (Å) and angles in degrees (°). E_1 and E_2 are excitation energies.

	C_4-C_5	C_5-C_6	$\angle N_1C_2N_3C_4$	$\angle C_4C_5C_6N_1$	E_1 (eV)	E_2 (eV)
CCSD	1.41	1.45	-30.5	-24.0	4.245448	4.272669
SCCSD	1.41	1.45	-30.5	-24.0	4.245379	4.272599

S_0/S_1 ε-MECIs for uracil

In Table 2 we report the values of some relevant internal coordinates for the ε-MECIs for uracil varying the value of ε. For the *oop*-O geometry, the ε-MECI was converged up to ε=0.005 Hartree. The $^6S_5$ ε-MECI was converged up to ε=0.0025 Hartree. For energy differences lower than this values, we encountered convergence issues of the ground state CCSD equations.

Table 2: Comparison of internal coordinates for the uracil MECIs. Distances are given in angstrom (Å) and angles in degrees (°). CCSD ε-MECIs were converged with the cc-pVDZ basis set. Ref. 1: SF-TDDFT/6-31+G(d,p). Ref. 2: CASSCF(10/8)/6-31G*.

	$^6S_5$					*oop*-O			
	CCSD			SF-TDDFT[1]	CASSCF[2]	CCSD			CASSCF[2]
ε (eV)	0.27	0.14	0.07			0.27	0.20	0.14	
C_4-O_8	1.21	1.21	1.21	1.20	1.19	1.41	1.42	1.42	1.52
C_5-C_6	1.46	1.46	1.46	1.43	1.45	1.35	1.35	1.35	1.34
C_4-C_5	1.49	1.49	1.49	1.47	1.49	1.48	1.48	1.48	1.46
$\angle C_4C_5C_6H_5$	113	114	114	121	112	179	179	179	179
$\angle C_6C_5C_4H_6$	21	22	22	24	21	1	1	1	1
$\angle C_4C_5C_6N_1$	50	51	52	53	52	1	1	0.5	1
$\angle C_2N_3C_4O_8$	163	165	166	161	-179	94	94	94	113
$\angle N_3C_4O_8$	119	119	119	119	118	85	85	85	82

S_0/S_1 MECI for azobenzene

Table 3: Comparison of internal coordinates for the azobenzene CI-rot MECI (6-31G). 5SA-CASSCF(6/6) results are taken from Ref. 3. The ε-MECI for CCSD was converged with $\varepsilon = 0.20$ eV. Distances are given in angstrom (Å) and angles in degrees (°).

	N_1-N_2	C_2-N_1	N_2-C_3	$\angle C_2N_1N_2C_3$	$\angle C_1C_2N_1N_2$	$\angle N_1N_2C_3C_4$	$\angle C_2N_1N_2$	$\angle N_1N_2C_3$
SA-CASSCF[3]	1.24	1.41	1.36	97	180	-15	122	149
CCSD	1.30	1.43	1.39	99	175	-13	119	134

References

(1) Zhang, X.; Herbert, J. M. Excited-State Deactivation Pathways in Uracil versus Hydrated Uracil: Solvatochromatic Shift in the 1 n π* State is the Key. *J. Phys. Chem. B* **2014**, *118*, 7806–7817.

(2) Nachtigallová, D.; Aquino, A. J.; Szymczak, J. J.; Barbatti, M.; Hobza, P.; Lischka, H. Nonadiabatic dynamics of uracil: Population split among different decay mechanisms. *J. Phys. Chem. A* **2011**, *115*, 5247–5255.

(3) Yu, L.; Xu, C.; Zhu, C. Probing the π π* photoisomerization mechanism of cis-azobenzene by multi-state ab initio on-the-fly trajectory dynamics simulation. *Phys. Chem. Chem. Phys.* **2015**, *17*, 17646–17660.
